# Click histochemistry for whole-mount staining of brain structures

**DOI:** 10.1016/j.mex.2019.09.011

**Published:** 2019-09-12

**Authors:** Alexander A. Lazutkin, Sergey A. Shuvaev, Natalia V. Barykina

**Affiliations:** a P.K. Anokhin Institute of Normal Physiology, 8 Baltiyskaya Str., 125315, Moscow, Russia; b Brain Stem Cell Laboratory, Moscow Institute of Physics and Technology, 9 Institutskiy Lane, 141701, Dolgoprudny, Moscow Region, Russia; c N.N. Burdenko Neurosurgery Institute, 13/5 1st Tverskoy-Yamskoy Lane, 125047, Moscow, Russia

**Keywords:** Whole-mount click histochemistry, Click chemistry, 5-ethynyl-2'-deoxyuridine, Whole-mount histochemistry, Neurogenesis, Cell division, Cell migration, Hippocampus, 3D imaging

## Abstract

Labeling of the replicating DNA with synthetic thymidine analogs is commonly used for marking the dividing cells. However, until now this method has only been applied to histological sections. A growing number of current approaches for three-dimensional visualization of large tissue samples requires detection of dividing cells within whole organs. Here we describe a method for labeling dividing cells with 5-ethynyl-2'-deoxyuridine (EdU) and their further detection in whole brain structures (for example, hippocampus) using the Cu (I) -catalyzed [3 + 2] cycloaddition reaction (so-called click-reaction). The presented method can be used for brain neurogenesis studies as well as for whole-mount staining of any preparations in which the terminal ethynyl group has been introduced.

• New click histochemistry method based on Cu (I) -catalyzed [3 + 2] cycloaddition reaction allows whole-mount staining of brain structures and other tissues.
• Our whole-mount click histochemistry method allows to visualize dividing cells in 3D and can be used in neurogenesis studies, i.e. for birthdating dividing early progenitors and further tracking of proliferation, survival, migration, differentiation, and fate of their progeny.
• Our whole-mount click histochemistry staining demonstrates high staining specificity, high signal intensity, and low background levels in young and adult mouse brain tissue.

## Graphical abstract

**Figure:**
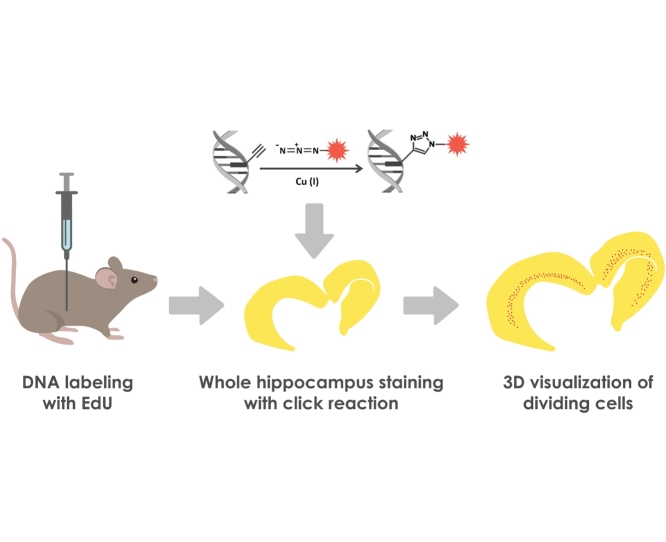

**Specification Table**

**Table:** 

Subject Area:	Neuroscience
More specific subject area:	Histochemistry, neurogenesis, 3D imaging
Method name:	Whole-mount click histochemistry
Name and reference of original method:	Click chemistry A. Salic, T.J. Mitchison, A chemical method for fast and sensitive detection of DNA synthesis in vivo, Proc. Natl. Acad. Sci. USA. 105 (2008) 2415-2420.
Resource availability:	NA

## Method details

A method described here allows staining whole brain structures using the Cu (I) -catalyzed [3 + 2] cycloaddition reaction (so-called click-reaction) between the terminal ethynyl group incorporated into DNA upon 5-ethynyl-2'-deoxyuridine (EdU) injection and fluorescently labeled azide. This method can be used in neurogenesis studies, i.e. for birthdating of dividing early progenitors and further tracking of proliferation, survival, migration, differentiation, and fate of their progeny. This whole-mount click method is based on the original protocol for staining tissue sections developed by Salic and Mitchison [**1**]. The following procedures were developed and optimized for whole hippocampus preparation staining and imaging.

### Step 1: tissue sample preparation

#### Materials

• 5-ethynyl-2’-deoxyuridine, 6.15 mg/ml
• 0.1 M phosphate buffer (PBS) pH 7.4
• 1% paraformaldehyde (PFA) in PBS, pH 7.4 at 4 °C
• 15% chloral hydrate
• 2 ml microtubes
• 1 ml syringes
• Mice were injected with a synthetic thymidine analogue 5-ethynyl-2’-deoxyuridine at a dose of 123 mg/kg 2 h before euthanasia. The time to euthanasia may vary depending on the specific research question. 2 h between analog injection and euthanasia is an often-used time interval to detect cells that have currently entered the S-phase of the cell cycle.
• Mice were anesthetized with chloral hydrate (10 mg/kg, Sigma, USA) immediately before euthanasia.
• Animals were intracardially perfused with 30 ml of PBS and 30 ml of cold 1% paraformaldehyde, pH 7.4.
• Brains were dissected 1 h after perfusion.
• The brain samples were postfixed by overnight (ON) immersion in 1% PFA at 4 °C.
• The cerebral cortex was removed from the fixed hemispheres using a microsurgical spatula, then the hippocampi were isolated and placed in 2 ml microtube with cold 1% PFA.
• Hippocampal specimens were additionally postfixed in 1% PFA, ON at 4 °C.
• Hippocampi were then rinsed twice with PBS for 2 h each time.

### Step 2: pretreatment and storage

#### Materials

• Eppendorf ThermoMixer Temperature Control Device (Eppendorf™ 5382000023)
• DMSO
• 100% methanol
• 50% methanol
• 25% methanol
• 12.5% methanol
• 30% H_2_O_2_ stock solution
• 10% Triton X-100 stock solution in PBS
• 10% saponin stock solution in PBS
• 0.1 M Tris-HCl buffer (pH 8.0)

All procedures described below were performed with constant stirring and at 20 °C, unless otherwise specified.

*Note:* Methanol requires careful handling since it is highly toxic and highly inflammable liquid. Microtubes must be filled fully to prevent the contact of specimens with air and development of autofluorescence on the samples’ surface.

(1) Specimens were treated in Dent solution (100% methanol/DMSO, in the ratio of 4:1) for 2 h at 4 °C.
(2) To destroy endogenous pigments, specimens were bleached in Dent bleach solution (100% methanol/DMSO/30% H_2_O_2_, in the ratio of 4:1:1) in bright light for 2 h until the samples are completely white.
(3) After bleaching, specimens were rinsed three times in 100% methanol for 1 h each time.
(4) Note: At this stage, specimens can be stored in 100% methanol at −70 °C. It is important to warm up the preparations gradually after storage to preserve the morphology. For this, the samples were first transferred to −20 °C, then to 4 °C, and only after that they were brought to room temperature.
(5) Samples were rehydrated stepwise in 50% methanol, 25% methanol, 12.5% methanol and PBS for 1 h in each solution.
(6) Rehydrated samples were washed twice in PBS for 1 h each time.
(7) Preparations were permeabilized in 2% saponin and 5% DMSO solution in PBS for 1 h at 37 °C.
(8) After permeabilization, the preparations were washed in Tris-HCl buffer (pH 8.0) with 0.2% Triton X-100 for 1 h.

### Step 3: click reaction

#### Materials

• DMSO
• 10% Triton X-100 stock solution in PBS
• 10% saponin stock solution in PBS
• 0.1 M Tris-HCl buffer (pH 8.0)
• 1 M sodium ascorbate stock solution (freshly prepared)
• 100 mM CuSO_4_ stock solution
• 1 mM fluorescent azide (e.g. Alexa Fluor 555 Azide, Triethylammonium Salt, A20012, Invitrogen) stock solution in DMSO
• 0.5 M EDTA (pH 8.0) stock solution

All procedures described below were performed with constant stirring and at 20 °C, unless otherwise specified.

*Note:* Click reaction solution must be prepared immediately before staining. 1 M sodium ascorbate stock solution must be prepared from the powder immediately before the click reaction.

(1) Specimens were incubated in click reaction solution (Tris−HCl buffer containing 5% DMSO, 0.2% Triton X-100, 0.2% saponin, 100 mM sodium ascorbate, 1 mM CuSO_4_ and 10 μM fluorescent azide) for 2 h.
(2) The click reaction was stopped by three 1 h incubations in Tris−HCl buffer with 5% DMSO, 0.2% Triton X-100 and 0.1 M EDTA.
(3) Stained preparations were washed twice for 1 h with PBS containing 5% DMSO and 0.2% Triton X-100 and twice with PBS for 1 h each time. Final rinsing with PBS was done overnight at 4 °C.

The whole-mount click histochemistry is compatible with other staining methods, for example, whole-mount immunohistochemistry [2,3] and whole-mount fluorescent Nissl staining [4] (data not shown, manuscript in preparation), with the second staining being performed after the click-reaction, following the protocols described previously [[2], [3], [4]].

### Step 4: clearing

#### Materials

• 25% ethanol
• 50% ethanol
• 96% ethanol
• 100% ethanol
• 2-butoxyethanol
• benzyl alcohol
• benzyl benzoate

All procedures described below were performed with constant stirring and at 20 °C, unless otherwise specified.

*Note:* Benzyl benzoate and benzyl alcohol require careful handling since they are strong non-polar solvents. Use only polyethylene and glass labware.

(1) The preparations were dehydrated in 25%, 50%, and 96% ethanol and three times in 100% ethanol for 1 h for each incubation.
(2) Dehydrated preparations were incubated in 2-butoxyethanol overnight. Specimens can be stored in 2-butoxyethanol for long time before and after clearing at 4 °C.
(3) Preparations were cleared for 12 h in Murray’s clearing solution (BBBA, benzyl benzoate and benzyl alcohol in the ratio of 2:1) in the dark at 4 °C.

Alternatively, preparations can be dehydrated in a series of methanol dilutions and cleared in dibenzyl ether as described [3,5]. A 30 min incubation with hexane can be added before clearing to achieve greater transparency of the samples.

### Step 5: imaging

The preparations were placed on a glass slide (from 2 to 6 pieces per glass) inside a square silicone frame. The frame was filled with a clarifying solution and covered with a coverslip, trying not to make an air bubble inside the liquid. The coverslip borders were outlined with a hydrophobic marker and distilled water was applied inside of these borders.

Imaging was performed using an upright laser scanning confocal microscope Olympus FluoView 1000 (Olympus) with water-immersion objective UM Plan FL N 20x / 0.50 W (Olympus). The imaging was carried out to the whole depth (up to 1.5 mm) of the specimen with a step of 5 μm, and 25–35 fields of view were stitched to create a three-dimensional reconstruction of the whole hippocampus. Stitching and 3D reconstruction were performed in Imaris 6.0 (Bitplane).

Alternatively, preparations can be imaged using light-sheet microscopy [6].

#### Method validation

To validate the method, we visualized dividing cells in the whole hippocampi of 2-week-, 2-month- and 12-month-old nestin-CFPnuc mice [7]. Dividing cells were labeled with EdU 2 h before euthanasia. Whole-mount click histochemistry was performed using Alexa Fluor 555 Azide (Invitrogen, A-20012). Overall, the protocol included bleaching (7 h), rehydration (6 h), permeabilization (3 h), staining (2 h), and rinsing (about 24 h), with the total duration of 4 days excluding the time required for sample preparation. The optical clearing procedures took additional 2 days.

**Fig. 1:**
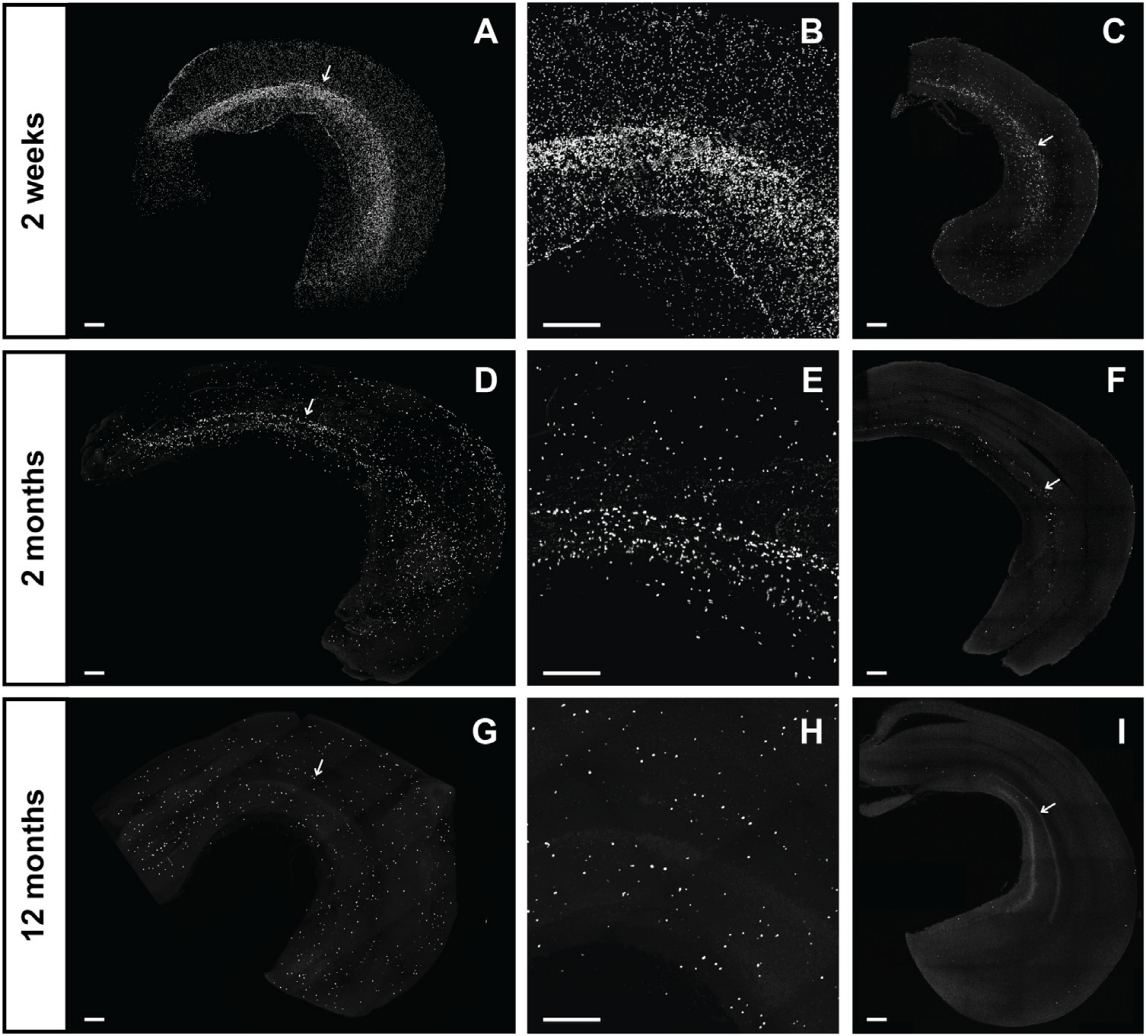
Dividing cells in whole hippocampi of 2-week-, 2-month- and 12-month-old mice. A,D,G and B,E,H – 3D reconstructions; C,F,I – optical sections. Arrows in A,D,G and C,F,I show the dentate gyrus; B,E,H - enlarged fragments of areas marked by arrows in A,D,G. Scales are 200 μm.

Examples of mouse whole-mount hippocampi stained using click histochemistry are presented in Fig. 1. Our whole-mount click histochemistry method achieved high staining specificity, high signal intensity, and low background levels both in young and adult hippocampi. Sparse dividing cells were found in all layers of hippocampus, while high labeled cell density was evident in the dentate gyrus, a brain area where neurogenesis occurs throughout life in rodents. The densities of dividing cell in the hippocampus decreased during normal aging, especially in the dentate gyrus.

We also validated our whole-mount click method for the quantitative analysis. In our previous study, an excellent signal-to-noise ratio of staining allowed to accurately detect labeled cells in the whole hippocampal volume using automatic cell counting algorithms [8].

The method described above was also used to reveal the effects of fast neutron irradiation on hippocampal cell proliferation. In the study, we combined our whole-mount click staining and quantitative 3D-analysis and showed suppressing of cell division along the entire dorsoventral axis of the hippocampus 24 h after the irradiation [9].

## Additional information

The study was carried out in accordance with the law of the Ministry of Health of the Russian Federation No. 267 from June 19, 2003 and according to protocol No. 1 from September 3, 2005 of the P.K. Anokhin Institute of Normal Physiology guidelines for animal experiments.
