# Supplementary material for: Click histochemistry for whole-mount staining of brain structures
Source: MethodsX. 2019 Sep 12;6:1986–91. doi: 10.1016/j.mex.2019.09.011 (PMC6812327; doi:10.1016/j.mex.2019.09.011)
Supplement: Supplementary file 1 [file mmc1.docx]

**Movie 1. Dividing cells in whole hippocampus of 2-week-old mice.**

**Movie 2. Dividing cells in whole hippocampus of 2-month-old mice.**

**Movie 3. Dividing cells in whole hippocampus of 12-month-old mice.**
